# (*N*′,*N*′′*Z*,*N*′,*N*′′*E*)-*N*′,*N*′′-[1-(4-Chloro­phen­yl)ethane-1,2-diyl­idene]bis­(3-methyl-1-benzofuran-2-carbohydrazide)

**DOI:** 10.1107/S1600536812030504

**Published:** 2012-07-10

**Authors:** Hoong-Kun Fun, Tze Shyang Chia, Ahmed M. Alafeefy, Hatem A. Abdel-Aziz

**Affiliations:** a X-ray Crystallography Unit, School of Physics, Universiti Sains Malaysia, 11800 USM, Penang, Malaysia; b Department of Pharmaceutical Chemistry, College of Pharmacy, Salman Bin Abdulaziz University, PO Box 173, Alkharj 11942, Saudi Arabia; c Department of Pharmaceutical Chemistry, College of Pharmacy, King Saud University, PO Box 2457, Riyadh 11451, Saudi Arabia

## Abstract

In the title compound, C_28_H_21_ClN_4_O_4_, the benzofuran ring systems make dihedral angles of 7.43 (8) and 30.92 (9)° with the chloro-substituted benzene ring. The dihedral angle between the two benzofuran ring systems is 27.41 (7)°. The two benzofuran rings are connected to the chloro-substituted benzene ring through C—N—N=C and C—N—N=C—C bridges which are nearly planar [maximum deviations = 0.003 (1) and 0.037 (1) Å]. An intra­molecular N—H⋯N hydrogen bond generates an *S*(6) ring motif. In the crystal, mol­ecules are linked by N—H⋯(O,N) and C—H⋯O hydrogen bonds into a tape along the *c* axis and these tapes are further connected by another weak C—H⋯O hydrogen bond into a sheet parallel to the *bc* plane. π–π inter­actions [centroid-to-centroid distances = 3.4845 (12)–3.6250 (13) Å] are also observed.

## Related literature

For the biological activity of benzofurans, see: Abdel-Aziz *et al.* (2009 ▶); Abdel-Aziz & Mekawey (2009 ▶); Abdel-Wahab *et al.* (2009 ▶); Bhovi *et al.* (2010 ▶). For the synthesis, see: Abdel-Aziz *et al.* (2009 ▶). For hydrogen-bond motifs, see: Bernstein *et al.* (1995 ▶). For the stability of the temperature controller used for the data collection, see: Cosier & Glazer (1986 ▶).
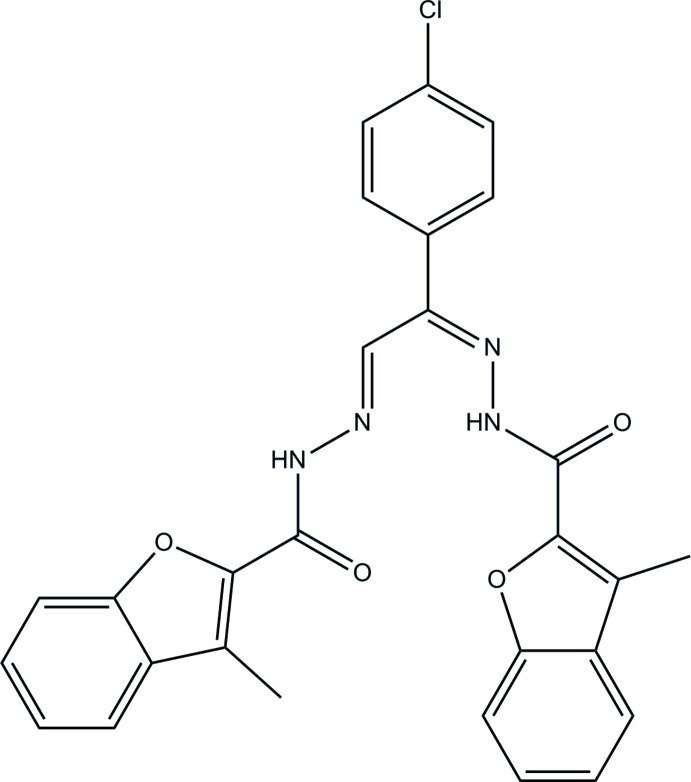


## Experimental

### 

#### Crystal data

C_28_H_21_ClN_4_O_4_
*M* *_r_* = 512.94
Monoclinic, 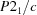

*a* = 7.5539 (9) Å
*b* = 23.332 (3) Å
*c* = 13.6027 (16) Å
β = 94.275 (2)°
*V* = 2390.8 (5) Å^3^
*Z* = 4
Mo *K*α radiation
μ = 0.20 mm^−1^
*T* = 100 K
0.30 × 0.11 × 0.09 mm

#### Data collection

Bruker APEX DUO CCD area-detector diffractometer
Absorption correction: multi-scan (*SADABS*; Bruker, 2009 ▶) *T*_min_ = 0.942, *T*_max_ = 0.983
19379 measured reflections
6850 independent reflections
4662 reflections with *I* > 2σ(*I*)
*R*_int_ = 0.043

#### Refinement

*R*[*F* ^2^ > 2σ(*F* ^2^)] = 0.048
*wR*(*F* ^2^) = 0.144
*S* = 1.03
6850 reflections
344 parameters
H atoms treated by a mixture of independent and constrained refinement
Δρ_max_ = 0.36 e Å^−3^
Δρ_min_ = −0.49 e Å^−3^

### 

Data collection: *APEX2* (Bruker, 2009 ▶); cell refinement: *SAINT* (Bruker, 2009 ▶); data reduction: *SAINT*; program(s) used to solve structure: *SHELXTL* (Sheldrick, 2008 ▶); program(s) used to refine structure: *SHELXTL*; molecular graphics: *SHELXTL*; software used to prepare material for publication: *SHELXTL* and *PLATON* (Spek, 2009 ▶).

## Supplementary Material

Crystal structure: contains datablock(s) global, I. DOI: 10.1107/S1600536812030504/is5163sup1.cif

Structure factors: contains datablock(s) I. DOI: 10.1107/S1600536812030504/is5163Isup2.hkl

Additional supplementary materials:  crystallographic information; 3D view; checkCIF report

## Figures and Tables

**Table 1:** Hydrogen-bond geometry (Å, °)

*D*—H⋯*A*	*D*—H	H⋯*A*	*D*⋯*A*	*D*—H⋯*A*
N1—H1N1⋯N3	0.89 (2)	1.96 (2)	2.644 (2)	132.3 (18)
N4—H1N4⋯O2^i^	0.95 (3)	2.31 (3)	3.187 (2)	154 (2)
N4—H1N4⋯N2^i^	0.95 (3)	2.48 (3)	3.205 (2)	134 (2)
C17—H17*A*⋯O2^i^	0.93	2.32	3.165 (2)	150
C28—H28*C*⋯O4^ii^	0.96	2.53	3.464 (2)	164

Symmetry codes: (i) 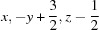; (ii) 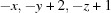.

**Fig. 1.**
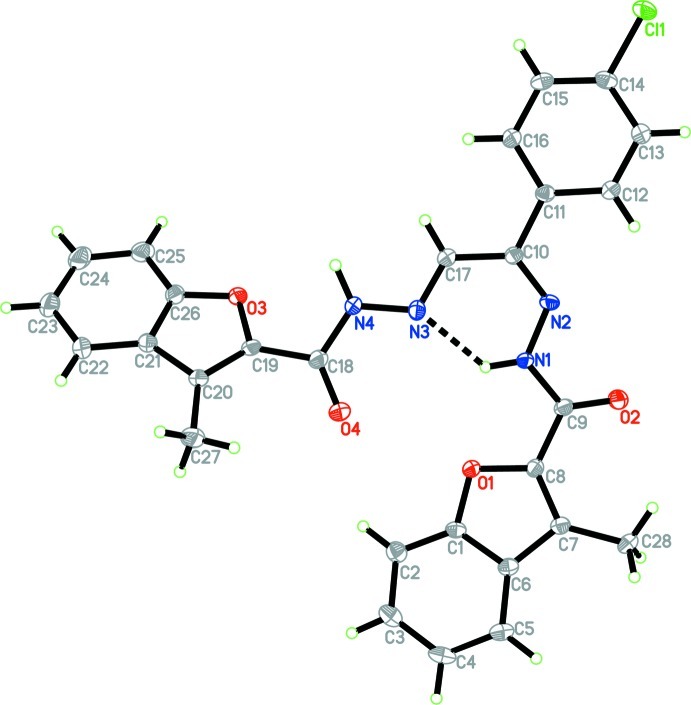
The molecular structure of the title compound with atom labels and 50% probability displacement ellipsoids. The dashed line represents the intramolecular N—H···N hydrogen bond.

**Fig. 2.**
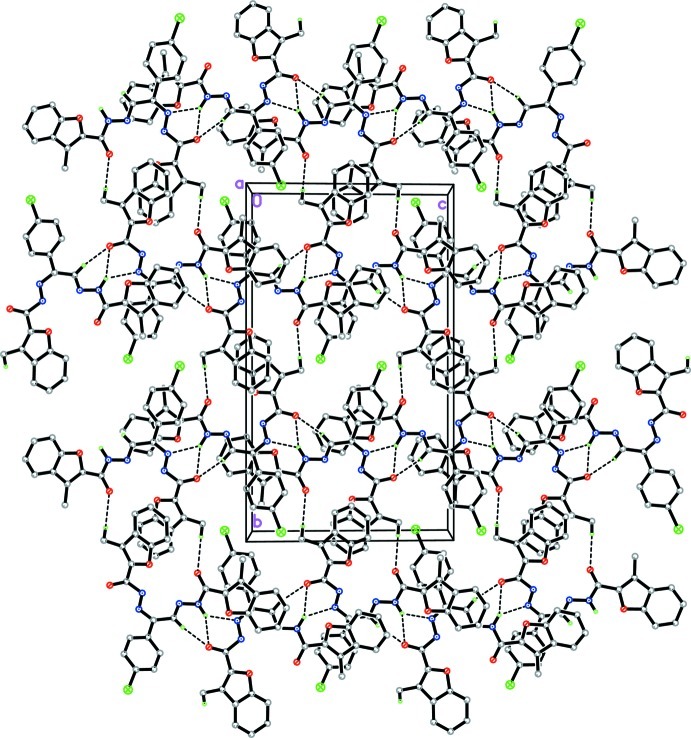
The crystal packing of the title compound. The dashed lines represent the hydrogen bonds. For sake of clarity, hydrogen atoms not involved in hydrogen bonding have been omitted.

## supplementary crystallographic information

### Comment

Benzofurans are found to be useful as anticonvulsant, anti-inflammatory, antitumor, antifungal, anthelmintic and antihyperglycemic agents (Abdel-Aziz *et al.*, 2009; Abdel-Aziz & Mekawey, 2009; Abdel-Wahab *et al.*, 2009; Bhovi *et al.*, 2010). In view of the biological activities and in continuation to our interest with benzofurans, we report herein the crystal structure of the title compound.

The molecular structure of the title compound is shown in Fig. 1. The O1/C1–C8 and O3/C19–C26 benzofuran ring systems (r.m.s. deviations = 0.0096 and 0.0052 Å, respectively) make dihedral angles of 7.43 (8) and 30.92 (9)°, respectively with the chloro-substituted C11–C16 benzene ring. The dihedral angle between the two benzofuran ring systems is 27.41 (7)°. The two benzofuran rings are connected to the chloro-substituted benzene ring through C9—N1—N2—C10 and C18—N4—N3—C17—C10 bridges which are nearly planar [maximum deviations = 0.003 (1) Å at atom N2 and 0.037 (1) Å at atom C17] and the ketone O2 and O4 atoms are almost coplanar with their attached bridges [N2—N1—C9—O2 = 0.1 (3)° and N3—N4—C18—O4 = 11.4 (3)°]. An intramolecular N1—H1N1···N3 hydrogen bond generates an S(6) ring motif (Bernstein *et al.*, 1995) in the molecule.

In the crystal (Fig. 2), the molecules are linked by N4—H1N4···(O2,N2) and C17—H17A···O2 hydrogen bonds (Table 1) into a tape along the *c* axis and the tapes are further connected by weak C28—H28C···O4 hydrogen bonds into a sheet parallel to the *bc* plane. π–π interactions are also observed with *Cg*1···*Cg*3 = 3.4845 (12) Å [-*x*, 2 - *y*, 1 - *z*], *Cg*2···*Cg*4 = 3.6250 (13) Å [-1 + *x*, 3/2 - *y*, -1/2 + *z*] and *Cg*3···*Cg*3 = 3.6124 (13) Å [-1 - *x*, 2 - *y*, 1 - *z*], where *Cg*1, *Cg*2, *Cg*3 and *Cg*4 are the centroids of O1/C1/C6–C8, O3/C19–C21/C26, C1–C6 and C11–C16 rings, respectively.

### Experimental

The title compound was prepared by the reaction of 3-methylbenzofuran-2-carbohydrazide with 2-chloro-1-(4-chlorophenyl)ethanone in absolute ethanol according to the reported method (Abdel-Aziz *et al.*, 2009). Colourless blocks suitable for an X-ray structural analysis were obtained by slow evaporation from EtOH/DMF.

### Refinement

Atoms H1N1 and H1N4 were located in a difference Fourier map and refined freely [N—H = 0.89 (2) and 0.95 (3) Å]. The remaining H atoms were positioned geometrically (C—H = 0.93 and 0.96 Å) and refined with *U*_iso_(H) = 1.2 or 1.5*U*_eq_(C). A rotating group model was applied to the methyl groups. An outlier, (011), was omitted in the final refinement.

### Crystal data

**Table:**

C_28_H_21_ClN_4_O_4_	*F*(000) = 1064
*M**_r_* = 512.94	*D*_x_ = 1.425 Mg m^−^^3^
Monoclinic, *P*2_1_/*c*	Mo *K*α radiation, λ = 0.71073 Å
Hall symbol: -P 2ybc	Cell parameters from 4684 reflections
*a* = 7.5539 (9) Å	θ = 2.3–29.8°
*b* = 23.332 (3) Å	µ = 0.20 mm^−^^1^
*c* = 13.6027 (16) Å	*T* = 100 K
β = 94.275 (2)°	Block, colourless
*V* = 2390.8 (5) Å^3^	0.30 × 0.11 × 0.09 mm
*Z* = 4	

### Data collection

**Table:**

Bruker APEX DUO CCD area-detector diffractometer	6850 independent reflections
Radiation source: fine-focus sealed tube	4662 reflections with *I* > 2σ(*I*)
Graphite monochromator	*R*_int_ = 0.043
φ and ω scans	θ_max_ = 29.9°, θ_min_ = 2.3°
Absorption correction: multi-scan (*SADABS*; Bruker, 2009)	*h* = −7→10
*T*_min_ = 0.942, *T*_max_ = 0.983	*k* = −32→28
19379 measured reflections	*l* = −18→18

### Refinement

**Table:**

Refinement on *F*^2^	Primary atom site location: structure-invariant direct methods
Least-squares matrix: full	Secondary atom site location: difference Fourier map
*R*[*F*^2^ > 2σ(*F*^2^)] = 0.048	Hydrogen site location: inferred from neighbouring sites
*wR*(*F*^2^) = 0.144	H atoms treated by a mixture of independent and constrained refinement
*S* = 1.03	*w* = 1/[σ^2^(*F*_o_^2^) + (0.0696*P*)^2^ + 0.8585*P*] where *P* = (*F*_o_^2^ + 2*F*_c_^2^)/3
6850 reflections	(Δ/σ)_max_ = 0.001
344 parameters	Δρ_max_ = 0.36 e Å^−^^3^
0 restraints	Δρ_min_ = −0.49 e Å^−^^3^

### Special details

**Table:**

Experimental. The crystal was placed in the cold stream of an Oxford Cryosystems Cobra open-flow nitrogen cryostat (Cosier & Glazer, 1986) operating at 100.0 (1) K.
Geometry. All e.s.d.'s (except the e.s.d. in the dihedral angle between two l.s. planes) are estimated using the full covariance matrix. The cell e.s.d.'s are taken into account individually in the estimation of e.s.d.'s in distances, angles and torsion angles; correlations between e.s.d.'s in cell parameters are only used when they are defined by crystal symmetry. An approximate (isotropic) treatment of cell e.s.d.'s is used for estimating e.s.d.'s involving l.s. planes.
Refinement. Refinement of *F*^2^ against ALL reflections. The weighted *R*-factor *wR* and goodness of fit *S* are based on *F*^2^, conventional *R*-factors *R* are based on *F*, with *F* set to zero for negative *F*^2^. The threshold expression of *F*^2^ > σ(*F*^2^) is used only for calculating *R*-factors(gt) *etc*. and is not relevant to the choice of reflections for refinement. *R*-factors based on *F*^2^ are statistically about twice as large as those based on *F*, and *R*-factors based on ALL data will be even larger.

### Fractional atomic coordinates and isotropic or equivalent isotropic displacement parameters (Å^2^)

**Table:**

	*x*	*y*	*z*	*U*_iso_*/*U*_eq_	
Cl1	0.45030 (7)	0.51063 (2)	0.65612 (4)	0.02517 (13)	
O1	−0.18297 (18)	0.91093 (5)	0.50974 (9)	0.0174 (3)	
O2	0.0296 (2)	0.84287 (6)	0.72023 (9)	0.0260 (3)	
O3	−0.26774 (18)	0.77075 (5)	0.10805 (9)	0.0182 (3)	
O4	−0.1284 (2)	0.88213 (6)	0.27065 (10)	0.0275 (3)	
N1	0.0177 (2)	0.81923 (6)	0.55642 (11)	0.0161 (3)	
N2	0.1004 (2)	0.76847 (6)	0.57851 (11)	0.0161 (3)	
N3	0.0059 (2)	0.78780 (7)	0.36930 (10)	0.0175 (3)	
N4	−0.0473 (2)	0.78768 (7)	0.27125 (11)	0.0190 (3)	
C1	−0.2346 (2)	0.96694 (8)	0.49610 (13)	0.0166 (4)	
C2	−0.3267 (3)	0.98844 (8)	0.41213 (14)	0.0207 (4)	
H2A	−0.3607	0.9653	0.3583	0.025*	
C3	−0.3650 (3)	1.04656 (9)	0.41339 (15)	0.0238 (4)	
H3A	−0.4277	1.0631	0.3591	0.029*	
C4	−0.3112 (3)	1.08106 (9)	0.49488 (15)	0.0242 (4)	
H4A	−0.3386	1.1199	0.4930	0.029*	
C5	−0.2186 (3)	1.05869 (8)	0.57782 (15)	0.0211 (4)	
H5A	−0.1820	1.0820	0.6310	0.025*	
C6	−0.1818 (2)	0.99973 (8)	0.57888 (13)	0.0168 (4)	
C7	−0.0928 (2)	0.96115 (8)	0.64901 (13)	0.0171 (4)	
C8	−0.0978 (2)	0.90913 (8)	0.60363 (12)	0.0167 (4)	
C9	−0.0134 (3)	0.85473 (8)	0.63364 (12)	0.0174 (4)	
C10	0.1336 (2)	0.73377 (7)	0.50707 (12)	0.0154 (3)	
C11	0.2211 (2)	0.67909 (8)	0.53963 (13)	0.0157 (3)	
C12	0.2970 (3)	0.67412 (8)	0.63637 (13)	0.0212 (4)	
H12A	0.3000	0.7059	0.6776	0.025*	
C13	0.3677 (3)	0.62277 (8)	0.67172 (14)	0.0225 (4)	
H13A	0.4167	0.6200	0.7363	0.027*	
C14	0.3648 (3)	0.57558 (8)	0.60979 (14)	0.0195 (4)	
C15	0.2966 (3)	0.57942 (8)	0.51350 (14)	0.0232 (4)	
H15A	0.2980	0.5477	0.4721	0.028*	
C16	0.2252 (3)	0.63126 (8)	0.47841 (14)	0.0205 (4)	
H16A	0.1796	0.6340	0.4132	0.025*	
C17	0.0845 (3)	0.74172 (8)	0.40184 (13)	0.0186 (4)	
H17A	0.1104	0.7130	0.3576	0.022*	
C18	−0.1258 (3)	0.83580 (8)	0.22966 (13)	0.0180 (4)	
C19	−0.2064 (2)	0.82603 (8)	0.12888 (13)	0.0168 (4)	
C20	−0.2279 (3)	0.86146 (8)	0.04978 (13)	0.0172 (4)	
C21	−0.3092 (3)	0.82657 (8)	−0.02879 (13)	0.0188 (4)	
C22	−0.3632 (3)	0.83616 (9)	−0.12821 (14)	0.0266 (4)	
H22A	−0.3489	0.8719	−0.1570	0.032*	
C23	−0.4381 (3)	0.79104 (10)	−0.18212 (15)	0.0328 (5)	
H23A	−0.4758	0.7966	−0.2481	0.039*	
C24	−0.4586 (3)	0.73683 (10)	−0.13934 (16)	0.0324 (5)	
H24A	−0.5101	0.7074	−0.1777	0.039*	
C25	−0.4041 (3)	0.72607 (9)	−0.04164 (15)	0.0257 (4)	
H25A	−0.4157	0.6901	−0.0132	0.031*	
C26	−0.3311 (2)	0.77218 (8)	0.01104 (13)	0.0189 (4)	
C27	−0.1779 (3)	0.92302 (8)	0.04235 (15)	0.0252 (4)	
H27A	−0.1037	0.9338	0.0997	0.038*	
H27B	−0.2833	0.9462	0.0380	0.038*	
H27C	−0.1146	0.9287	−0.0155	0.038*	
C28	−0.0066 (3)	0.97664 (8)	0.74798 (14)	0.0230 (4)	
H28A	0.0764	0.9473	0.7696	0.034*	
H28B	−0.0956	0.9802	0.7944	0.034*	
H28C	0.0549	1.0124	0.7435	0.034*	
H1N1	−0.013 (3)	0.8287 (9)	0.4940 (17)	0.023 (6)*	
H1N4	−0.033 (3)	0.7535 (12)	0.2352 (19)	0.043 (7)*	

### Atomic displacement parameters (Å^2^)

**Table:**

	*U*^11^	*U*^22^	*U*^33^	*U*^12^	*U*^13^	*U*^23^
Cl1	0.0302 (3)	0.0151 (2)	0.0296 (2)	0.00422 (19)	−0.00130 (19)	0.00252 (17)
O1	0.0233 (7)	0.0133 (6)	0.0152 (6)	−0.0004 (5)	−0.0006 (5)	−0.0009 (5)
O2	0.0435 (9)	0.0199 (7)	0.0143 (6)	0.0074 (6)	0.0013 (6)	0.0017 (5)
O3	0.0223 (7)	0.0133 (6)	0.0182 (6)	−0.0003 (5)	−0.0025 (5)	−0.0016 (5)
O4	0.0436 (9)	0.0151 (7)	0.0226 (7)	0.0026 (6)	−0.0047 (6)	−0.0031 (5)
N1	0.0245 (8)	0.0101 (7)	0.0135 (7)	0.0008 (6)	−0.0003 (6)	−0.0001 (5)
N2	0.0192 (8)	0.0098 (7)	0.0191 (7)	0.0006 (6)	0.0004 (6)	0.0005 (5)
N3	0.0210 (8)	0.0174 (8)	0.0138 (7)	−0.0005 (6)	−0.0016 (6)	−0.0002 (5)
N4	0.0260 (9)	0.0158 (8)	0.0145 (7)	0.0032 (6)	−0.0027 (6)	−0.0025 (6)
C1	0.0168 (9)	0.0122 (8)	0.0209 (8)	−0.0009 (7)	0.0028 (7)	0.0002 (6)
C2	0.0196 (9)	0.0201 (10)	0.0222 (9)	−0.0016 (7)	0.0005 (7)	0.0025 (7)
C3	0.0198 (10)	0.0225 (10)	0.0289 (10)	0.0013 (8)	0.0005 (8)	0.0067 (8)
C4	0.0219 (10)	0.0138 (9)	0.0375 (11)	0.0032 (7)	0.0059 (8)	0.0039 (8)
C5	0.0197 (9)	0.0147 (9)	0.0293 (10)	−0.0006 (7)	0.0040 (8)	−0.0036 (7)
C6	0.0162 (9)	0.0149 (9)	0.0196 (8)	0.0002 (7)	0.0034 (7)	−0.0011 (6)
C7	0.0188 (9)	0.0143 (9)	0.0182 (8)	−0.0005 (7)	0.0022 (7)	−0.0011 (6)
C8	0.0207 (9)	0.0150 (9)	0.0143 (7)	−0.0012 (7)	0.0002 (7)	−0.0007 (6)
C9	0.0227 (10)	0.0133 (9)	0.0165 (8)	−0.0010 (7)	0.0029 (7)	−0.0017 (6)
C10	0.0161 (9)	0.0128 (8)	0.0169 (8)	−0.0015 (6)	−0.0006 (6)	−0.0005 (6)
C11	0.0147 (9)	0.0135 (8)	0.0188 (8)	−0.0010 (7)	0.0011 (6)	−0.0010 (6)
C12	0.0251 (10)	0.0177 (9)	0.0201 (8)	0.0024 (8)	−0.0033 (7)	−0.0046 (7)
C13	0.0264 (11)	0.0205 (10)	0.0197 (9)	0.0041 (8)	−0.0031 (7)	−0.0011 (7)
C14	0.0195 (9)	0.0132 (9)	0.0258 (9)	0.0008 (7)	0.0021 (7)	0.0018 (7)
C15	0.0301 (11)	0.0136 (9)	0.0257 (9)	0.0020 (8)	−0.0002 (8)	−0.0047 (7)
C16	0.0259 (10)	0.0164 (9)	0.0187 (8)	0.0005 (7)	−0.0022 (7)	−0.0019 (7)
C17	0.0231 (10)	0.0156 (9)	0.0168 (8)	0.0009 (7)	−0.0003 (7)	−0.0026 (6)
C18	0.0218 (9)	0.0151 (9)	0.0171 (8)	−0.0010 (7)	0.0014 (7)	−0.0016 (6)
C19	0.0199 (9)	0.0118 (8)	0.0183 (8)	−0.0003 (7)	−0.0005 (7)	−0.0029 (6)
C20	0.0190 (9)	0.0141 (9)	0.0184 (8)	0.0020 (7)	0.0007 (7)	−0.0009 (6)
C21	0.0198 (9)	0.0192 (10)	0.0172 (8)	0.0038 (7)	0.0005 (7)	−0.0011 (7)
C22	0.0363 (12)	0.0248 (11)	0.0181 (9)	0.0076 (9)	−0.0025 (8)	−0.0002 (7)
C23	0.0401 (13)	0.0350 (13)	0.0217 (9)	0.0093 (10)	−0.0093 (9)	−0.0075 (8)
C24	0.0343 (13)	0.0308 (12)	0.0300 (11)	0.0039 (9)	−0.0110 (9)	−0.0124 (9)
C25	0.0247 (10)	0.0205 (10)	0.0308 (10)	0.0002 (8)	−0.0049 (8)	−0.0062 (8)
C26	0.0171 (9)	0.0202 (9)	0.0187 (8)	0.0022 (7)	−0.0017 (7)	−0.0027 (7)
C27	0.0329 (12)	0.0168 (10)	0.0259 (9)	−0.0006 (8)	0.0031 (8)	0.0012 (7)
C28	0.0298 (11)	0.0191 (10)	0.0196 (8)	0.0000 (8)	−0.0007 (8)	−0.0060 (7)

### Geometric parameters (Å, º)

**Table:**

Cl1—C14	1.7465 (19)	C11—C16	1.394 (2)
O1—C1	1.372 (2)	C11—C12	1.400 (2)
O1—C8	1.387 (2)	C12—C13	1.383 (3)
O2—C9	1.230 (2)	C12—H12A	0.9300
O3—C26	1.370 (2)	C13—C14	1.386 (3)
O3—C19	1.392 (2)	C13—H13A	0.9300
O4—C18	1.217 (2)	C14—C15	1.374 (3)
N1—N2	1.362 (2)	C15—C16	1.394 (3)
N1—C9	1.372 (2)	C15—H15A	0.9300
N1—H1N1	0.89 (2)	C16—H16A	0.9300
N2—C10	1.304 (2)	C17—H17A	0.9300
N3—C17	1.291 (2)	C18—C19	1.476 (2)
N3—N4	1.364 (2)	C19—C20	1.357 (2)
N4—C18	1.372 (2)	C20—C21	1.444 (3)
N4—H1N4	0.95 (3)	C20—C27	1.491 (3)
C1—C2	1.386 (3)	C21—C26	1.395 (3)
C1—C6	1.395 (2)	C21—C22	1.401 (3)
C2—C3	1.387 (3)	C22—C23	1.380 (3)
C2—H2A	0.9300	C22—H22A	0.9300
C3—C4	1.405 (3)	C23—C24	1.406 (3)
C3—H3A	0.9300	C23—H23A	0.9300
C4—C5	1.385 (3)	C24—C25	1.385 (3)
C4—H4A	0.9300	C24—H24A	0.9300
C5—C6	1.403 (3)	C25—C26	1.384 (3)
C5—H5A	0.9300	C25—H25A	0.9300
C6—C7	1.441 (2)	C27—H27A	0.9600
C7—C8	1.361 (2)	C27—H27B	0.9600
C7—C28	1.496 (2)	C27—H27C	0.9600
C8—C9	1.465 (3)	C28—H28A	0.9600
C10—C17	1.463 (2)	C28—H28B	0.9600
C10—C11	1.489 (2)	C28—H28C	0.9600
			
C1—O1—C8	105.12 (13)	C15—C14—Cl1	120.19 (15)
C26—O3—C19	105.10 (14)	C13—C14—Cl1	118.78 (14)
N2—N1—C9	117.26 (14)	C14—C15—C16	119.41 (17)
N2—N1—H1N1	120.4 (14)	C14—C15—H15A	120.3
C9—N1—H1N1	122.3 (14)	C16—C15—H15A	120.3
C10—N2—N1	119.09 (15)	C15—C16—C11	121.00 (17)
C17—N3—N4	115.35 (15)	C15—C16—H16A	119.5
N3—N4—C18	119.22 (15)	C11—C16—H16A	119.5
N3—N4—H1N4	118.1 (16)	N3—C17—C10	121.18 (16)
C18—N4—H1N4	122.6 (16)	N3—C17—H17A	119.4
O1—C1—C2	125.17 (16)	C10—C17—H17A	119.4
O1—C1—C6	110.60 (15)	O4—C18—N4	124.01 (16)
C2—C1—C6	124.24 (17)	O4—C18—C19	122.89 (17)
C1—C2—C3	115.84 (18)	N4—C18—C19	113.10 (15)
C1—C2—H2A	122.1	C20—C19—O3	112.62 (15)
C3—C2—H2A	122.1	C20—C19—C18	131.24 (17)
C2—C3—C4	121.44 (18)	O3—C19—C18	116.11 (15)
C2—C3—H3A	119.3	C19—C20—C21	105.23 (16)
C4—C3—H3A	119.3	C19—C20—C27	128.68 (17)
C5—C4—C3	121.71 (18)	C21—C20—C27	126.09 (16)
C5—C4—H4A	119.1	C26—C21—C22	119.06 (18)
C3—C4—H4A	119.1	C26—C21—C20	106.37 (15)
C4—C5—C6	117.75 (18)	C22—C21—C20	134.56 (19)
C4—C5—H5A	121.1	C23—C22—C21	118.0 (2)
C6—C5—H5A	121.1	C23—C22—H22A	121.0
C1—C6—C5	119.00 (17)	C21—C22—H22A	121.0
C1—C6—C7	106.45 (16)	C22—C23—C24	121.32 (19)
C5—C6—C7	134.54 (17)	C22—C23—H23A	119.3
C8—C7—C6	105.12 (15)	C24—C23—H23A	119.3
C8—C7—C28	128.27 (17)	C25—C24—C23	121.77 (19)
C6—C7—C28	126.54 (16)	C25—C24—H24A	119.1
C7—C8—O1	112.71 (16)	C23—C24—H24A	119.1
C7—C8—C9	130.51 (16)	C26—C25—C24	115.7 (2)
O1—C8—C9	116.43 (15)	C26—C25—H25A	122.1
O2—C9—N1	123.33 (17)	C24—C25—H25A	122.1
O2—C9—C8	122.71 (16)	O3—C26—C25	125.22 (18)
N1—C9—C8	113.93 (15)	O3—C26—C21	110.68 (16)
N2—C10—C17	126.98 (16)	C25—C26—C21	124.09 (17)
N2—C10—C11	114.60 (15)	C20—C27—H27A	109.5
C17—C10—C11	118.30 (15)	C20—C27—H27B	109.5
C16—C11—C12	117.99 (17)	H27A—C27—H27B	109.5
C16—C11—C10	122.49 (16)	C20—C27—H27C	109.5
C12—C11—C10	119.47 (16)	H27A—C27—H27C	109.5
C13—C12—C11	121.25 (17)	H27B—C27—H27C	109.5
C13—C12—H12A	119.4	C7—C28—H28A	109.5
C11—C12—H12A	119.4	C7—C28—H28B	109.5
C12—C13—C14	119.25 (17)	H28A—C28—H28B	109.5
C12—C13—H13A	120.4	C7—C28—H28C	109.5
C14—C13—H13A	120.4	H28A—C28—H28C	109.5
C15—C14—C13	121.03 (17)	H28B—C28—H28C	109.5
			
C9—N1—N2—C10	−179.44 (17)	C12—C13—C14—C15	1.5 (3)
C17—N3—N4—C18	−177.48 (17)	C12—C13—C14—Cl1	−178.68 (16)
C8—O1—C1—C2	−179.32 (19)	C13—C14—C15—C16	−1.7 (3)
C8—O1—C1—C6	0.6 (2)	Cl1—C14—C15—C16	178.55 (16)
O1—C1—C2—C3	−179.65 (18)	C14—C15—C16—C11	−0.3 (3)
C6—C1—C2—C3	0.5 (3)	C12—C11—C16—C15	2.4 (3)
C1—C2—C3—C4	0.6 (3)	C10—C11—C16—C15	−175.19 (18)
C2—C3—C4—C5	−0.4 (3)	N4—N3—C17—C10	−174.21 (16)
C3—C4—C5—C6	−0.9 (3)	N2—C10—C17—N3	3.1 (3)
O1—C1—C6—C5	178.29 (16)	C11—C10—C17—N3	178.91 (17)
C2—C1—C6—C5	−1.8 (3)	N3—N4—C18—O4	11.4 (3)
O1—C1—C6—C7	−0.6 (2)	N3—N4—C18—C19	−169.09 (16)
C2—C1—C6—C7	179.31 (18)	C26—O3—C19—C20	−0.2 (2)
C4—C5—C6—C1	2.0 (3)	C26—O3—C19—C18	−178.47 (16)
C4—C5—C6—C7	−179.5 (2)	O4—C18—C19—C20	31.0 (3)
C1—C6—C7—C8	0.4 (2)	N4—C18—C19—C20	−148.4 (2)
C5—C6—C7—C8	−178.3 (2)	O4—C18—C19—O3	−151.14 (19)
C1—C6—C7—C28	177.63 (19)	N4—C18—C19—O3	29.4 (2)
C5—C6—C7—C28	−1.0 (4)	O3—C19—C20—C21	−0.1 (2)
C6—C7—C8—O1	0.0 (2)	C18—C19—C20—C21	177.8 (2)
C28—C7—C8—O1	−177.21 (18)	O3—C19—C20—C27	−179.37 (18)
C6—C7—C8—C9	172.82 (19)	C18—C19—C20—C27	−1.5 (4)
C28—C7—C8—C9	−4.4 (4)	C19—C20—C21—C26	0.4 (2)
C1—O1—C8—C7	−0.4 (2)	C27—C20—C21—C26	179.71 (19)
C1—O1—C8—C9	−174.26 (16)	C19—C20—C21—C22	−178.8 (2)
N2—N1—C9—O2	0.1 (3)	C27—C20—C21—C22	0.5 (4)
N2—N1—C9—C8	178.22 (15)	C26—C21—C22—C23	0.8 (3)
C7—C8—C9—O2	24.7 (3)	C20—C21—C22—C23	180.0 (2)
O1—C8—C9—O2	−162.71 (18)	C21—C22—C23—C24	−0.6 (3)
C7—C8—C9—N1	−153.5 (2)	C22—C23—C24—C25	−0.3 (4)
O1—C8—C9—N1	19.1 (2)	C23—C24—C25—C26	0.9 (3)
N1—N2—C10—C17	−2.9 (3)	C19—O3—C26—C25	179.39 (19)
N1—N2—C10—C11	−178.84 (15)	C19—O3—C26—C21	0.5 (2)
N2—C10—C11—C16	162.87 (18)	C24—C25—C26—O3	−179.46 (19)
C17—C10—C11—C16	−13.4 (3)	C24—C25—C26—C21	−0.8 (3)
N2—C10—C11—C12	−14.7 (3)	C22—C21—C26—O3	178.77 (17)
C17—C10—C11—C12	169.01 (17)	C20—C21—C26—O3	−0.6 (2)
C16—C11—C12—C13	−2.5 (3)	C22—C21—C26—C25	−0.1 (3)
C10—C11—C12—C13	175.12 (18)	C20—C21—C26—C25	−179.49 (19)
C11—C12—C13—C14	0.6 (3)		

### Hydrogen-bond geometry (Å, º)

**Table:**

*D*—H···*A*	*D*—H	H···*A*	*D*···*A*	*D*—H···*A*
N1—H1*N*1···N3	0.89 (2)	1.96 (2)	2.644 (2)	132.3 (18)
N4—H1*N*4···O2^i^	0.95 (3)	2.31 (3)	3.187 (2)	154 (2)
N4—H1*N*4···N2^i^	0.95 (3)	2.48 (3)	3.205 (2)	134 (2)
C17—H17*A*···O2^i^	0.93	2.32	3.165 (2)	150
C28—H28*C*···O4^ii^	0.96	2.53	3.464 (2)	164

Symmetry codes: (i) *x*, −*y*+3/2, *z*−1/2; (ii) −*x*, −*y*+2, −*z*+1.
